# Ecological Balance in Unmanaged Beech Reserves: Scolytids or Their Natural Saproxylic Beetle Enemies?

**DOI:** 10.3390/insects16111087

**Published:** 2025-10-23

**Authors:** Václav Zumr, Oto Nakládal, Lukáš Bílek, Jiří Remeš

**Affiliations:** Faculty of Forestry and Wood Sciences, Czech University of Life Sciences Prague, Kamýcká 129, Suchdol, 165 00 Prague, Czech Republic; nakladal@fld.czu.cz (O.N.); bilek@fld.czu.cz (L.B.); remes@fld.czu.cz (J.R.)

**Keywords:** conservation, biological control, deadwood, bark beetle

## Abstract

**Simple Summary:**

Beech reserves with a high accumulation of dead wood may potentially host large populations of scolytid beetles. Currently, several non-native scolytid species are quite rapidly spreading across Europe. However, a high diversity of dead wood also supports numerous saproxylic beetles that can act as effective natural regulators of scolytid populations. In our study, a high number of natural enemies among saproxylic beetles was recorded, significantly exceeding the abundance of scolytids. This finding suggests that beech reserves represent relatively stable biocenoses, maintaining internal balance through natural interactions.

**Abstract:**

The accumulation of dead wood can serve as a potential source of insect pests, with scolytids being the most frequently discussed group. The aim of this study was to quantify the abundance and species composition of scolytids and their natural saproxylic beetle enemies in a beech reserve. In addition, we compared the types of dead wood preferred by scolytids and their natural enemies. Beetles were sampled passively using window traps, which effectively capture the actual density of beetles within the stand. In total, 20,515 saproxylic beetles were collected; the analyses included 11 scolytids species with 3017 individuals and 51 species of natural enemies with 4976 individuals. The results revealed a significantly higher abundance of natural saproxylic beetle enemies, with no strong affiliation to specific types of dead wood. This may indicate a high mobility of natural enemies actively searching for food resources within the forest stands. In conclusion, beech reserves support high abundances of natural scolytid enemies that exceed the numbers of scolytids themselves, indicating effective natural control processes.

## 1. Introduction

Due to the ongoing decline in species diversity [1], one of the fundamental strategies to support diverse and rare habitats is the establishment of unmanaged forest areas [2]. Currently, integrated forest management [3] or a mosaic of different management approaches at the landscape scale [2] are also coming to the fore. One of the objectives is to abandon management practices and thereby stimulate the creation of attributes similar to those found in old-growth forests, which are now almost entirely absent in their original state in Europe [4,5]. Beech (*Fagus sylvatica* L.) is one of the most widespread broadleaved tree species in Europe; however, over the past centuries, extensive substitution by coniferous trees has occurred [6], resulting in a significantly reduced distribution of beech compared to its original range. Beech reserves are relatively common elements in the landscape, as they provide a substantial number of microhabitats, e.g., [7]. They also contain an important component—dead wood—which supports a variety of organisms, including saproxylic insects, soil fauna, and other invertebrates [8,9,10,11]. Dead wood is also crucial for the habitat of wood-decaying fungi [12] and provides nesting sites for cavity-dwelling birds and bats [13,14]. Reserves are generally established in areas where significant species have survived as relicts, typically species listed under the Habitats Directive (Council Directive 92/43/EEC). Typical old-growth beech forest species include *Rhysodes sulcatus* (Fabricius, 1787), *Rosalia alpina* (Linnaeus, 1758), or *Cucujus cinnaberinus* (Scopoli, 1763). Protection is also aimed at preserving original refugia of entire biocenoses within beech stands, generally corresponding to *Luzulo-Fagetum* and *Asperulo-Fagetum* forest types (Council Directive 92/43/EEC). However, newly established unmanaged areas can potentially pose a threat by allowing harmful beetle species to proliferate. Recent beech reserves generally maintain their standard structure–even-aged classes. Once a certain age is reached, however, the stands relatively quickly undergo decay [15]. Beech is a relatively short-lived tree species and stands begin to decompose after approximately 200–300 years [6]. Such sudden accumulation of fresh dead wood can lead to population outbreaks of potentially harmful agents.

The subfamily Curculionidae: Scolytinae represents the main concern. In the Czech Republic, this subfamily comprises around 122 species [16], compared to 107 species recorded across Czechoslovakia in the 1950s [17]. However, not all species are harmful, as many are indifferent or neutral. Economically significant species, primarily associated with coniferous trees, include, for example, *Ips typographus* (Linnaeus, 1758) and *Pityogenes chalcographus* (Linnaeus, 1760) on spruce [17]. Outbreaks of these species can create large areas of dead wood [18], but besides the negative economic impacts, positive ecological responses among other insects have also been observed, e.g., [19]. Overall, concerns regarding bark beetle outbreaks include both economic damage to forestry operations [20] and threats such as wildfires, particularly in protected areas where logging does not occur [21]. A notable example is the largest forest fire in 2022 (1200 ha) in the Czech Republic, which occurred in the Bohemian Switzerland National Park [22].

Beech hosts very few harmful beetle or insect species. For example, the forestry entomology textbook by Křístek [23] lists only five insect pests associated with beech (Coleoptera: *Agrilus viridis* (Linnaeus, 1758) (Coleoptera: Buprestidae), *Calliteara pudibunda* Linnaeus, 1758 (Lepidoptera: Erebidae), *Operophtera fagata* (Scharfenberg, 1805) (Lepidoptera: Geometridae), *Cryptococcus fagisuga* Lindinger, 1936 (Hemiptera: Eriococcidae) and *Phyllaphis fagi* (Linnaeus, 1761) (Hemiptera: Aphididae), compared to 41 species reported for spruce. Nevertheless, beech can host several polyphagous bark beetle species, and notably, it is potentially vulnerable to new threats in the form of invasive scolytids. The introduction of invasive pests continues at a rapid pace. Over the past decade, several dozen new bark beetle species have been recorded in Europe [24]. Among them, *Xylosandrus germanus* (C.H. Hoffmann, 1941) is the most widespread invasive bark beetle in Europe [25], primarily attacking beech as its host [26]. This species can be effectively monitored using ethanol-baited traps [27].

Bark beetles have a range of natural enemies, including birds, but particularly other invertebrates such as Coleoptera, Hymenoptera, Diptera, and even Nematoda [17,28]. Structural complexity is a key factor in creating habitats for these antagonistic species [28]. Saproxylic beetles are abundant in beech reserves [29,30], and many of these species feed on bark beetles at various developmental stages. Examples include *Nemozoma elongatulum* (Linnaeus, 1760) [31], *Colydium* sp., *Rhizophagus* sp., *Epuraea* sp., members of the Cucujidae, as well as beetles from the families Carabidae (mainly genus *Tachyta* sp., *Dromius* sp.) and Staphylinidae [17]. Many of these natural enemies are so closely associated with their prey, in this case bark beetles, that they can detect the prey’s pheromones [32].

The aim of this study was to assess whether recent beech reserves are capable of supporting outbreaks of harmful bark beetle species or, conversely, whether certain types of dead wood provide higher abundances of antagonistic beetle species that feed on at least one developmental stage of bark beetles.

## 2. Materials and Methods

### 2.1. Study Site

The study was conducted in the forest area of the Voděradské bučiny (49.96° N, 14.79° E), located in the Czech Republic (Central Europe). The area (682 ha) represents an extensive complex of old-growth beech stands, serving as a refugium for submontane to montane plant, fungal, and animal species. Unlike many other surrounding forest complexes, the area was permanently covered with forest. The elevation ranges from 345 to 501 m a.s.l. Climate: average annual precipitation is about 650 mm, and mean annual temperature is around 8 °C. Relief: The terrain is hilly, featuring a number of periglacial formations such as pseudokarst, boulder streams, and polygonal soils. Pedology: The dominant soil type is Cambisol. The oldest historical sources (land registers) indicate that beech forests predominated here, with a significant presence of fir trees [33,34]. The current beech stands regenerated naturally through a shelterwood system between 1800 and 1820. Since 1955, the area has been excluded from conventional forest management with a slow, controlled transition to non-intervention. Today, over sixty percent of the reserve is allocated for spontaneous development, as outlined in the Care Plan [34].

### 2.2. Studied Beetle Groups

The study focused exclusively on the group of saproxylic beetle antagonists of bark beetles (Curculionidae: Scolytinae; hereafter referred to as scolytids). Captured beetles were identified to the species level and classified as either saproxylic or non-saproxylic according to [35], since any species feeding on scolytid beetles is considered saproxylic. Ulyshen [36] defined saproxylic insects as those depending, either directly or indirectly, on dying or dead wood. Directly dependent species consume parts of woody tissues (i.e., bark, phloem, or wood), whereas indirectly dependent species feed on other wood-dependent organisms (e.g., wood-decaying fungi or other saproxylic taxa) or use dead wood as nesting substrate. From the set of saproxylic beetles, species were classified as scolytid antagonists at any developmental stage based on available information on their bionomy [17,31,37]. A comprehensive overview of predatory relationships among beetles is provided by Horák & Nakládal [32]. Scolytids were designated as potential pest species, including both obligate and facultative beech-associated taxa [17]. Due to the trapping method and identification constraints, the families Carabidae and Staphylinidae were not included, as they are typically monitored using pitfall traps [38]. Staphylinidae were excluded because of the high difficulty of determination and the lack of specialists, although they represent an important group of natural scolytid enemies [17]. However, their species and abundance patterns are expected to correlate closely with other saproxylic antagonists, showing similar environmental responses [39]. Some beetle families that are more difficult to identify have been identified by specialists: Jan Horák (Praha): Scraptiidae, Mordelidae; Pavel Průdek (Brno): Cerylonidae, Ciidae, Corylophagidae, Cryptophagidae, Latridiidae, Monotomidae, Zopheridae; Josef Jelínek (Praha): Nitidulidae. The taxonomy of species followed Zich O. (ed.) (2022), BioLib (http://www.biolib.cz (accessed on 10 September 2025)).

### 2.3. Beetle Sampling

The data were collected over a three-year period (2021–2023). Standard window traps without attractants were used, capturing entomological material passively. More detailed information about the trap design is provided in [29,40]. Window traps effectively record the actual density of beetles moving within forest stands and the local species pool [41], in contrast to ethanol-baited traps, which may bias results due to their long-distance attraction. Traps were installed at permanent sampling plots to ensure consistent stand conditions across multiple collection events. The degree of wood decay was kept relatively uniform to allow reliable comparisons among individual samples [29,40]. In total, eighty-nine traps were used. Thirty traps were installed in each of the first two years, while twenty-nine were deployed in 2023, resulting in eighty-nine traps used throughout the study. The traps were active from April to September and were checked regularly every 2–3 weeks. In 2021, traps were mounted on poles at a height of 1.5 m, evenly distributed across the plots as a control. In 2022, traps were attached directly to standing beech snags, oriented southward, one trap per trunk, at a height of 1.5 m. In 2023, traps were placed directly on lying logs, positioned approximately 5 cm above the ground using small supports. Elevating the traps prevented epigeic organisms from directly entering the collecting cups. The preservative solution consisted of propylene glycol (1:1.5 with water) with a drop of detergent to reduce surface tension.

### 2.4. Analysis

Species numbers and individuals count, divided into scolytids and their enemies, were used as response variables, with each trap considered as an independent sampling unit. The fixed factor was the type of dead wood position, including control traps. Overall differences between the studied factors were tested using generalized linear models (GLMs) with likelihood ratio chi-square tests implemented in the car::Anova package [42] and the glmmTMB package [43]. The Conway-Maxwell-Poisson distribution, parameterized with the exact mean [44], was applied because it is flexible enough to accommodate both overdispersed and underdispersed count data [43]. Individual trap IDs were used as random factor. Also, all models were checked for overdispersion using the “DHARMa” package [45].

To evaluate the accumulation and completeness of species richness (γ diversity) for both scolytids and their enemies, we followed the approach of Chao & Jost [46], which allows less biased comparisons of species richness between communities and requires a lower sampling effort. Hill numbers (q = 0 species richness) were calculated as proposed by Chao et al. [47]. These calculations were performed to evaluate sample coverage, performed in the iNEXT package with incidence-based species data and 50 bootstrap replications [48]. These analyses were performed in R version 4.3.1 (R Core Team) [49].

Patterns of community composition and their relationships were examined using unconstrained ordination–Detrended Correspondence Analysis (DCA) following Šmilauer & Lepš [50]. This approach provides an overview of species composition structure [50]. Log-transformed abundance data input were used as input, and detrending by segments was applied (gradient length of the first axis: 2.74). The output included species scatterplots (visualizing all scolytid species and enemy species with >10 individuals) and sample positions. The analysis was performed in CANOCO 5 [50]. Indicator Species Analysis (IndVal) [51] was used to identify species significantly associated with particular factors. This analysis was conducted using the “indicspecies” package, applying the “multipatt” function with duleg = TRUE [52]. The significance level was set at of α = 0.05.

Finally, correlations between the numbers and abundances of scolytids and their natural enemies were analyzed using a second-degree polynomial regression with natural log-transformation of abundance data, implemented in the Statistica program.

## 3. Results

**Figure 1:**
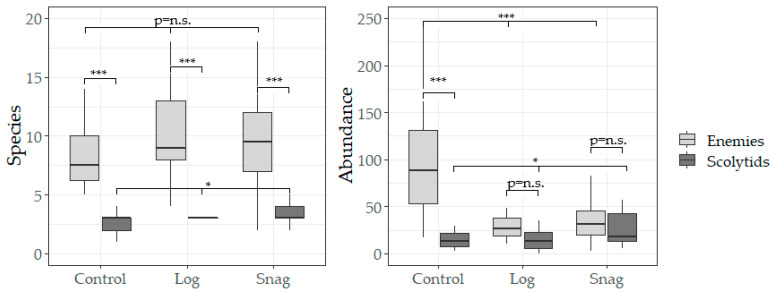
Numbers of species and individuals per trap for the studied beetle groups. Boxes indicate the interquartile range (Q1–Q3), the solid line within each box represents the median, and the error lines show min–max values. Asterisks above the bars indicate significant differences (* <0.05; *** <0.001; n.s. = nonsignificant).

**Figure 2:**
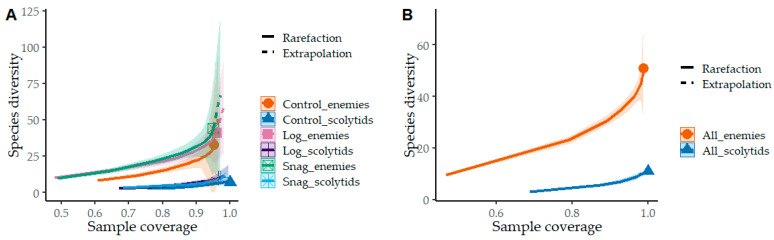
Sample coverage-based rarefaction and extrapolation of species richness (q = 0). Richness of scolytids and enemies of saproxylic beetles among the studied factors (**A**). Total species coverage at the studied site (**B**). Solid points indicate observed sample coverage, with the number of recorded species divided according to the studied factors and beetle groups. Dashed lines (**A**) indicate extrapolated values, and shaded areas represent 95% confidence intervals.

**Figure 3:**
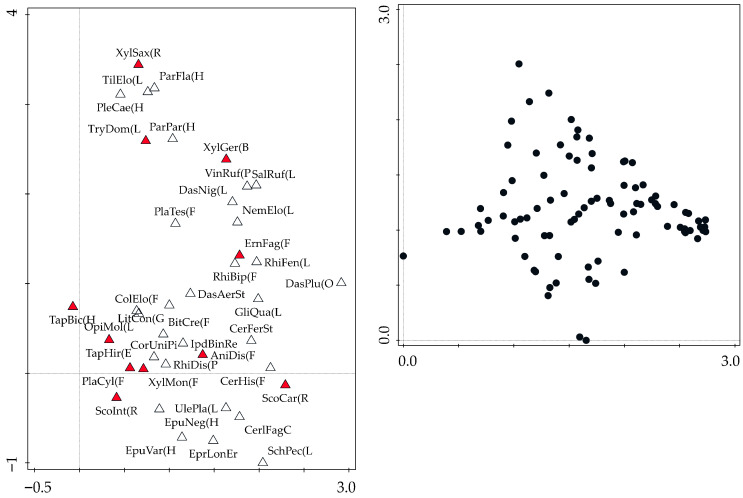
DCA ordination scatterplot showing species (in red: all scolytids + enemies with >10 individuals, displayed within the transparent triangle) and sample position. The first two axes are used in both plots. Abbreviations are shown in Table 1.

**Figure 4:**
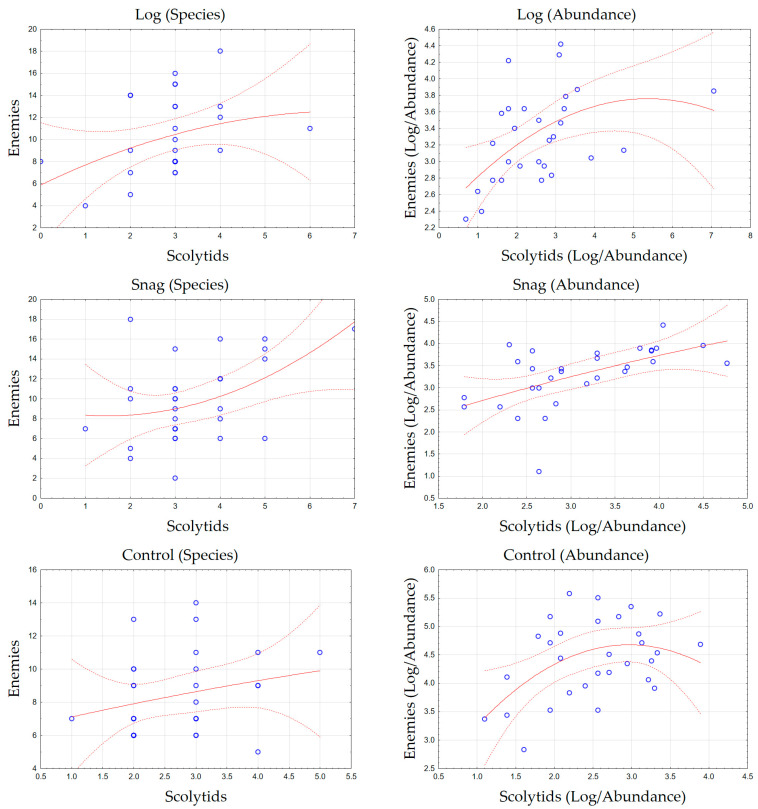
Polynomial regression showing the relationship between scolytids and their natural enemies based on log-transformed abundance and species richness across the studied factors.

Based on three-year research, a total of 20,515 individuals were collected, among 311 saproxylic beetle species were identified. Of these, 11 scolytid species comprising 3017 individuals were analyzed, along with 51 species of natural enemies totaling 4976 individuals. The most abundant scolytid was *Taphrorychus bicolor* (Herbst, 1793), while *Dasytes plumbeus* (Müller, 1776) represented the most numerous natural enemy. The overall numbers of species and individuals per trap are shown in Figure 1. The distribution of species richness was relatively balanced among the studied factors and beetle groups, whereas abundance patterns differed more distinctly. No significant differences in beetle abundance were found between logs and snags; however, higher numbers of natural enemies were recorded in the control plots, while scolytids were slightly more abundant in snags. Gamma diversity revealed similar trends—natural enemies were represented by markedly more species than scolytids (Figure 2A). Nevertheless, a complete spectrum of saproxylic beetle species (100% sample coverage) was documented for both groups (Figure 2B). The structural ecological composition of species was illustrated using Detrended Correspondence Analysis (DCA; Figure 3). The analysis indicated no strong clustering of species (first two axes explained 31.65% of the variation), suggesting high habitat connectivity. Relationships between the two beetle groups were examined using polynomial regression (Figure 4). Abundance data showed significant correlations: logs (r = 0.43, *p* = 0.020), snags (r = 0.56, *p* = 0.001), and control plots (r = 0.38, *p* = 0.038). A similar pattern was observed for species richness: logs (r = 0.32, *p* = 0.084), snags (r = 0.43, *p* = 0.018), and controls (r = 0.25, *p* = 0.178). Species preferences showed only weak associations with the studied factors (Table 1). In total, 16 species exhibited direct affiliations, of which only one was a scolytid.

## 4. Discussion

In this study, several beech-associated scolytid species were recorded as potential pests, with the capacity to increase in abundance in the studied reserve, where the original stands are currently undergoing relatively rapid decay. Over the sampling period using passive methods, the analysis [46] revealed a complete community of scolytids as well as their natural enemies among saproxylic beetles. However, saproxylic beetles represent only a part of the complex network of scolytid antagonists. A substantial proportion of the natural enemies includes Formicidae, parasitic Hymenoptera, and Diptera [37,53], while entomopathogenic fungi and birds also play important roles [54]. Other insect orders and beetle families, such as Carabidae and Staphylinidae, which may also represent significant natural enemies, were not included in this study [17]. Some studies, e.g., Parisi et al. [55], report a high number (54% of the total) of Staphylinidae specimens in beech forests. However, not all species of this family are enemies of scolytids [17].

Interestingly, the numbers of species and individuals across the studied forest variables were generally similar. However, control plots yielded higher abundances, primarily due to the predominance of *Dasytes plumbeus* (Melyridae). A similarly high abundance of Melyridae was found in the traps placed higher up by Bouget et al. [56]. This suggests that these natural enemies are relatively mobile within the stands and occur in substantial numbers.

Horák & Nakládal [32] report, for some species, the consumption of scolytid eggs and larvae: *Thanasimus formicarius* consumes over 50 larvae and more than 100 adult scolytids over its lifetime; *Rhizophagus dispar* consumes 15 eggs per adult per day; *Rhizophagus ferrugineus*, 79 eggs per adult over its lifespan; *Epuraea marseuli*, 23 eggs per day; *Epuraea pygmaea*, 9 eggs per day; *Corticeus fraxini*, 93 larvae per larval lifetime; *Nemozoma elongatum* consumes 2 adults per day and over 40 larvae, pupae, and immature scolytid beetles per larva over its lifespan. These numbers indicate relatively high feeding demands for some of the species captured in this study. For context, Pfeffer [17] notes that female scolytids generally lay one egg per day, although total fecundity varies by species—from as few as 10–15 eggs in the genus *Trypophloeus* to over one hundred eggs in *Ips typographus* [17]. Nevertheless, the precise trophic habits and consumption rates of eggs and larvae for most predatory saproxylic species remain largely unknown. The spectrum of natural enemies recorded in this study also highlights substantial overlap in habitat use with the scolytids. Overall, it is evident that maintaining high species richness of saproxylic organisms requires variability and sufficient volumes of dead wood [57], along with adequate sunlight [58]. Also, semi-natural beech stands support large numbers of saproxylic beetles [29], and correspondingly, low-intensity forest management increases both scolytid mortality and predation pressure [59].

The freshness of dead wood is also important. Scolytids typically colonize the freshest wood, while many potential natural enemies prefer wood at slightly more advanced stages of decay. These predators can subsequently move through the stand and consume various life stages of scolytid beetles. The pattern observed in this study suggests that higher numbers of natural enemies are active within the stands. The study area contains substantial volumes of dead wood [60], although still less than in long-term forest reserves [61]. Overall, the total amount of dead wood in the studied area may influence scolytid abundances by providing resources for natural enemies, while also affecting the rate of beech wood decomposition. Beech wood decomposes faster than other tree species [62].

The most abundant scolytid beetles were *Taphrorychus bicolor*, *Xylosandrus germanus*, and *Xyleborinus saxesenii*. Other species were highly marginal in terms of their abundance, likely due to their facultative association with beech, which limited their numbers. *T. bicolor* is a phloeophagous species, whereas *X. germanus* and *X. saxesenii* are both ambrosia beetles. These species are common inhabitants of beech forests. The first-mentioned species is native, and traps baited with attractants can capture tens of thousands of individuals without any signs of forest damage [63]. Holuša et al. [63] reported that *T. bicolor* prefers short-distance dispersal, and its abundance can rapidly increase in beech stands weakened by drought. The highest abundance of this species corresponds to the fact that the accumulation of dead wood may enhance its population density. In contrast, *X. germanus* is a non-native scolytid that occurs widely and contributes significantly to the flying beetle biomass in beech forests, as observed in this study. This aligns with its status as the most abundant non-native scolytid in Europe [25]. Gossner et al. [60] highlighted the high variability of *X. germanus* populations across different sites. The abundance of invasive *X. germanus* may be increased by management [60]. We can confirm this fact from our unpublished data from other locations, which show that commonly managed stands host high numbers of this species. In the first year (unpublished data), the same number of individuals *X. germanus* was caught using the same type of traps (n = 30) compared to in this three-year study in unmanaged beech stands. This may correspond to [28], which shows that decreasing habitat structure significantly diminished natural enemy abundance. In our study, despite the three-year duration, most species did not show significant fluctuations in abundance, nor did they differ according to dead wood type. Scolytids generally have one or more generations per year [17]; thus, if there had been exceptionally high abundances in the study area, this would have been reflected in increased trap captures.

Feeding specialization plays a key role in determining a species’ occupancy of a given habitat. When essential requirements are not met, species are unable to establish or persist. In our study, saproxylic beetles largely depend on microhabitats within dead wood during their larval stage, exhibiting diverse trophic strategies, such as carnivory and fungivory. Adults, however, often leave dead wood and primarily feed on flowering plants, which can act as a significant limiting factor in managed forests [64]. The availability of scolytid beetles as prey may further enhance the overall abundance of saproxylic beetles in beech forest reserves.

## 5. Conclusions

Beech forest reserves, even those established decades ago, do not appear to pose a risk of large-scale scolytid beetle outbreaks. Instead, our study reveals that these habitats support a diverse and abundant community of natural enemies, particularly saproxylic beetles. This highlights the dual role of small-scale reserves within managed forests: not only do they enhance overall biodiversity, but they also contribute to natural pest regulation, demonstrating that conservation and forest health are tightly interconnected. The results suggest that beech reserves function as resilient biocenoses, capable of maintaining ecological stability over time. Nevertheless, much remains to be explored regarding the complex interactions between natural enemies and scolytid beetles.

## Figures and Tables

**Table 1:** Captured species and their association with the studied factors. Species highlighted in bold were identified as significant indicators species for the respective factors according to the IndVal method (α < 0.05; [51]).

		Log	Snag	Control
Scolytids (Sum)	Abbreviations	1665	897	455
*Anisandrus dispar* (Fabricius, 1792)	*AniDis(F*	3	1	2
*Ernoporicus fagi* (Fabricius, 1798)	*ErnFa(F*	16	21	23
*Platypus cylindrus* (Fabricius, 1792)	*PlaCyl(F*	2	3	
*Scolytus carpini* (Ratzeburg, 1837)	*ScoCar(R*	1	1	2
*Scolytus intricatus* (Ratzeburg, 1837)	*ScoInt(R*	**3**		
*Taphrorychus bicolor* (Herbst, 1793)	*TapBic(H*	1466	476	191
*Taphrorychus hirtellus* (Eichhoff, 1878)	*TapHir(E*		1	
*Trypodendron domesticum*(Linnaeus, 1758)	*TryDom(L*	4	18	6
*Xyleborinus saxesenii* (Ratzeburg, 1837)	*XylSax(R*	17	**111**	
*Xyleborus monographus* (Fabricius, 1792)	*XylMon(F*	11	4	2
*Xylosandrus germanus* (Blandford, 1894)	*XylGer(B*	142	261	229
Enemies (Sum)		908	958	3110
*Aplocnemus impressus* (Marsham, 1802)	*AplImp(M*		1	
*Aplocnemus nigricornis* (Fabricius, 1792)	*AplNig(F*			2
*Bitoma crenata* (Fabricius, 1775)	*BitCre(F*	10	16	7
*Cerylon deplanatum* (Gyllenhal, 1827)	*CerDep(G*		2	
*Cerylon fagi* C.N.F.Brisout de Barneville, 1867	*CerFag(C*	**44**	5	21
*Cerylon ferrugineum* Stephens, 1830	*CerFer(S*	61	58	78
*Cerylon histeroides* (Fabricius, 1792)	*CerHis(F*	30	17	**53**
*Colydium elongatum* (Fabricius, 1787)	*ColElo(F*	8	7	3
*Corticeus bicolor* (Olivier, 1790)	*CorBic(O*	1	3	
*Corticeus fraxini* (Kugelann, 1794)	*CorFra(K*		1	
*Corticeus unicolor* Piller & Mitterpacher, 1783	*CorUni(P*	38	64	20
*Cucujus cinnaberinus* (Scopoli, 1763)	*CucCin(S*		4	
*Dasytes aeratus* Stephens, 1829	*DasAer(S*	4	6	1
*Dasytes niger* (Linnaeus, 1761)	*DasNig(L*	1	6	7
*Dasytes plumbeus* (O. F. Müller, 1776)	*DasPlu(O*	206	163	**2420**
*Epuraea distincta* (Grimmer, 1841)	*EpuDis(G*		1	
*Epuraea longula* Erichson, 1845	*EpuLon(E*	**27**	1	1
*Epuraea marseuli* Reitter, 1872	*EpuMar(R*	4	1	
*Epuraea neglecta* (Heer, 1841)	*EpuNeg(H*	**15**	1	
*Epuraea pallescens* (Stephens, 1830)	*EpuPal(S*	1	1	2
*Epuraea pygmaea* (Gyllenhal, 1808)	*EpuPyg(G*		1	
*Epuraea variegata* (Herbst, 1793)	*EpuVar(H*	**50**	3	
*Glischrochilus quadripunctatus* (Linnaeus, 1758)	*GliQua(L*	3	9	1
*Ipidia binotata* Reitter, 1875	*IpiBin(R*	10	11	3
*Litargus connexus* (Geoffroy in Fourcroy, 1785)	*LitCon(G*	27	26	11
*Malachius bipustulatus* (Linnaeus, 1758)	*MalBip(L*	1		
*Nemozoma elongatum* (Linnaeus, 1761)	*NemElo(L*	12	22	**47**
*Opilo mollis* (Linnaeus, 1758)	*OpiMol(L*	3	7	
*Paromalus flavicornis* (Herbst, 1792)	*ParFla(H*	2	**48**	1
*Paromalus parallelepipedus* (Herbst, 1792)	*ParPar(H*	1	**8**	1
*Pediacus depressus* (Herbst, 1797)	*PedDep(H*	**3**		
*Placonotus testaceus* (Fabricius, 1787)	*PlaTes(F*	5	6	
*Platysoma compressum* (Herbst, 1783)	*PlaCom(H*	1		1
*Plegaderus caesus* (Herbst, 1792)	*PleCae(H*	1	**21**	1
*Pyrochroa coccinea* (Linnaeus, 1761)	*PyrCoc(L*	2	1	6
*Rhizophagus bipustulatus* (Fabricius, 1792)	*RhiBip(F*	151	195	200
*Rhizophagus cribratus* (Gyllenhal, 1827)	*RhiCri(G*	1		
*Rhizophagus dispar* (Paykull, 1800)	*RhiDis(P*	20	13	3
*Rhizophagus fenestralis* (Linnaeus,1758)	*RhiFen(L*	2	4	5
*Rhizophagus ferrugineus* (Paykull, 1800)	*RhiFer(P*		1	
*Rhizophagus nitidulus* (Fabricius, 1798)	*RhiNit(F*	6	2	1
*Rhizophagus perforatus* Erichson, 1845	*RhiPer(E*	**5**	1	
*Salpingus planirostris* (Fabricius, 1787)	*SalPla(F*		1	4
*Salpingus ruficollis* (Linnaeus, 1761)	*SalRuf(L*	4	6	5
*Schizotus pectinicornis* (Linnaeus, 1758)	*SchPec(L*	**32**	1	9
*Silvanoprus fagi* (Guérin-Ménéville, 1844)	*SilFag(G*	1		
*Silvanus unidentatus* (Olivier, 1790)	*SilUni(O*			1
*Thanasimus formicarius* (Linnaeus, 1758)	*ThaFor(L*	1	4	1
*Tillus elongatus* (Linnaeus, 1758)	*TilElo(L*	9	**111**	1
*Uleiota planatus* (Linnaeus, 1761)	*UlePla(L*	**21**	4	9
*Vincenzellus ruficollis* (Panzer, 1794)	*VinRuf(P*	84	94	184

## Data Availability

The original contributions presented in this study are included in the article. Further inquiries can be directed to the corresponding author.
